# Epidemiology of pain in back and extremities in rural population: A community-based estimation of age- and sex-specific prevalence, distribution, duration and intensity of pain, number of painful sites and seasonality of pain during twelve months in rural Gadchiroli, India

**DOI:** 10.7189/jogh.11.12002

**Published:** 2021-11-27

**Authors:** Anand A Bang, Shekhar Y Bhojraj, Mahesh Deshmukh, Vinay R Joshi, Tushar Yermal, Sameer Kalkotwar, Abhay T Bang

**Affiliations:** 1 Society for Education, Action and Research in Community Health (SEARCH), Gadchiroli, Maharashtra, India; 2 Spine Foundation, Mumbai, Maharashtra, India; 3 Hinduja Hospital and Research Center, Mumbai, Maharashtra, India; 4 Naraindas Morbai Budhrani Trust, Mumbai, Maharashtra, India

## Abstract

**Background:**

Population-based estimates of the burden of pain in back and extremities (PBE) by sex, age, intensity, seasonality and site are lacking from rural India.

**Methods:**

Two villages were randomly selected from a cluster of 39 villages in Gadchiroli district in India. All residents’≥20 years of age were surveyed in January 2010 by trained surveyors by making household visits. Information on PBE in the 12 months prior to survey was obtained using a structured, pretested questionnaire.

**Results:**

The 12-month period prevalence of PBE was 75% (95% confidence interval CI = 72.54-77.73) in men and 91% (95% CI = 88.66-92.13) in women. The prevalence of PBE in the participants >50 years was 94% while that in the age group 20 to 50 years was 79% (*P* < 0.05). The site with the highest prevalence of pain was low back (women 80%, men 59%). The mean number of painful sites per person was 5.42 (95% CI = 5.17-5.67) in women, 3.68 (95% CI = 3.45-3.90) in men, 3.89 (95% CI = 3.71-4.07) in participants aged 20 to 50 years and 6.48 (95% CI = 6.11-6.85) in those >50 years. Among participants across the age and sex groups, the prevalence of mild pain was higher than severe pain at all the anatomical sites. Among various seasons, the highest prevalence of pain was in the rainy season (14%).

**Conclusion:**

The prevalence and the number of painful sites were higher among women and in those >50 years of age. The public health interventions for PBE need to focus on these two high risk groups.

Back pain (BP) and musculoskeletal pain (MSP) are the commonest form of chronic pain, causing disability and health care expenditure world over [1-4]. Heavy physical work is a known risk factor for back and musculoskeletal pain [5] and hence agrarian rural communities across the world are at a high risk of BP and MSP [6-8]. Nevertheless, though there was significant data available on the epidemiology of BP and MSP from the developed countries, the data from the developing countries and especially rural and agrarian communities was lacking. To fill this gap, the World Health Organization (WHO) and the International League Against Rheumatism (ILAR) launched a joint initiative called COPCORD (Community Oriented Program for the Control of Rheumatic Diseases). The aim was to conduct population based surveys using uniform methods, focusing on recording symptoms instead of diagnosing diseases. Starting from the Philippines, COPCORD has contributed enormously over the years in providing data from several countries from Asia, South and Central America, and Egypt on the burden and epidemiology of BP and MSP [9].

However, population-based data on the epidemiology of the burden of back and musculoskeletal pain from rural India, focusing on communities which almost exclusively are agrarian and rely on manual labour is lacking, thereby also limiting the possibility of developing appropriate interventions.

This study, estimating the age and sex specific burden of pain in back and extremities (PBE) in the adult (≥20 years) population in rural Gadchiroli in the Maharashtra state in India fills this gap. The burden was measured as, age- and sex-specific a) 12-month period prevalence, b) site specific prevalence, c) number of painful sites per adult, d) duration of pain, e) the intensity of pain and f) seasonality of pain in the rural adult population in Gadchiroli, India, over a period of 12 months.

## METHODS

### Study design and sample size

This study was nested in a population-based, cross-sectional survey of the prevalence of PBE in rural Gadchiroli. The study setting, study design, detail method of sample size calculation, method of village selection including the eligibility criteria are described in detail elsewhere [10].

### Questionnaire development, training, quality control and data collection

A questionnaire in vernacular language (Marathi) was developed to interview the participants and record the information about: a) episodes of pain in back and extremities at different body sites, b) duration of each pain episode and c) intensity of pain at each site in the 12 months preceding January 2010. The questionnaire was pilot tested in villages that were not part of the study and in the rural clinic of SEARCH. Community health workers (CHWs) were trained in administering the questionnaire and the data were collected from 1 January 2010 to 25 January 2010, details of which are described elsewhere [10].

### Statistical methods

A database was constructed for data entry using FOX PRO Version 2.0 (Microsoft Inc, Redmond, Washington, USA). The data were double entered, validated and checked for inconsistencies. Descriptive statistics included mean, medians and ranges for continuous variables and proportions for categorical variables were calculated. The age and sex specific 12-month period prevalence and the number of painful sites were estimated with their associated 95% confidence intervals (CI). Student’s *t* test was used for comparison of means. Differences between proportions were assessed using Chi-sqaure test. We followed STROBE guidelines for the reporting of observational studies. Analyses were conducted using Stata 10.0 (Stata Corp, College Station, Texas, USA).

### Ethical approval

The research followed the tenets of the Declaration of Helsinki. Ethical approval for this nested study was granted as part of the main study, by the Institutional Ethical Committee of SEARCH formed according to the guidelines by the Indian Council for Medical Research. Consent was obtained first at the cluster level in the study villages 15 days before starting the survey. The community leaders (Village Council Leaders and members, school-teacher and presidents of microfinance self-help groups) were explained the purpose and scope of the study including the benefits to the villagers (availability of referral care in SEARCH clinic and the care through a village clinic). Informed written consent in vernacular language in a standard format was obtained from individual participants after explaining the nature and benefits of the study. The benefits provided during the study included free consultation by spine surgeons and rheumatologists in a clinic conducted in the same village at a later date. For those who needed further evaluation, laboratory investigations, as well as imaging with Magnetic Resonance Imaging (MRI) and x-ray including transport were provided free of cost. For patients needing pharmacotherapy and physiotherapy, these services were provided free of cost and for those needing surgical interventions, such services were provided at significantly subsidized costs. The CHWs discussed these benefits using a printed pamphlet.

## RESULTS

**Figure 1:**
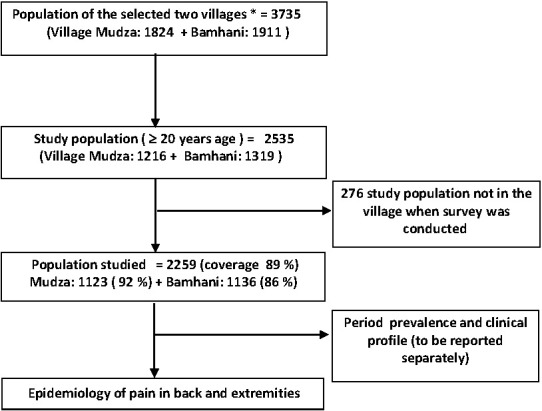
Study design flowchart. *2010 population register.

The total population of the two villages was 3735 out of which 2535 (67.9%) were adults ≥20 years of age and were eligible to participate in the study (**Figure 1**). Of these, 2259 (89%) were interviewed, 276 (11%) were either absent from the village (migrated for work) or unable to communicate due to very old age or disability. Total 1101 men (49%) and 1158 (51%) women participated in the study. Proportion of illiteracy was higher in women (55%) participants than men (22.2%). The demographic characteristics of the participants are described in **Table 1**.

**Table 1:** Socio-demographic characteristics of the population studied by sex (n = 2259)

Characteristic	Men (n = 1101)*		Women (n = 1158)†		Total	
	**n**	**%**	**n**	**%**	**n**	**%**
**Total**	1101	49	1158	51	2259	
**Caste:**						
Schedule castes	100	9.1	115	9.9	215	9.5
Schedule tribes	146	13.3	172	14.9	318	14.1
Other castes	855	77.7	871	75.2	1726	76.4
**Education (years):**						
Illiterate	244	22.2	643	55.5	887	39.3
1-4	264	24.0	131	11.3	395	17.5
5-7	163	14.8	111	9.6	274	12.1
8-10	272	24.7	200	17.3	472	20.9
11-12	128	11.6	62	5.4	190	8.4
>12	30	2.7	11	0.9	41	1.8
Mean education (standard deviation)	6 (4.3)		4 (4.4)		5 (4.5)	
**Age (years):**						
20-30	353	32.1	325	28.1	678	30.0
31-40	229	20.8	287	24.8	516	22.8
41-50	222	20.2	249	21.5	471	20.8
51-60	146	13.3	155	13.4	301	13.3
>60	151	13.7	142	12.3	293	13.0
Mean age (standard deviation)	41.6 (15.8)		41.8 (15.3)		41.7 (15.6)	
**Occupation:**						
Labour	471	42.8	528	45.6	999	44.2
Farmer	375	34.1	390	33.7	765	33.9
Service	44	4.0	25	2.2	69	3.1
Household work	93	8.4	100	8.6	193	8.5
Business	79	7.2	78	6.7	157	6.9
Other	39	3.5	37	3.2	76	3.4

*1108 men out of total 1216 men in village.

†1158 women out of total 1319 women in village.

### Prevalence according to sex and age group

**Figure 2:**
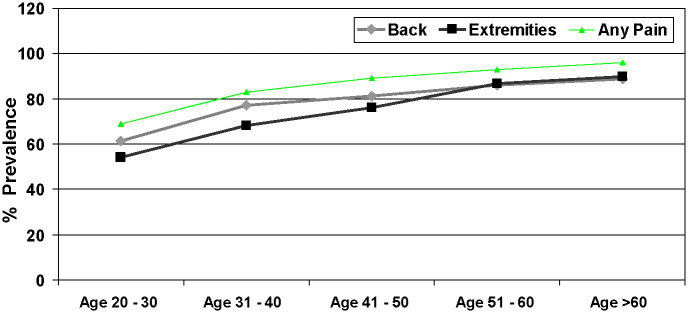
Prevalence of pain in back and extremities by age group (January 2009 to January 2010, n = 2259). Categories are multiple and overlapping.

The 12-month period prevalence of back pain was 66% (95% CI = 63.05-68.73) in men and 86% (95% CI = 83.42-87.55) in women, of pain in the extremities was 63% (95% CI = 60.19-65.98) in men and 78% (95% CI = 75.83-80.66) in women and of any back/extremity pain was 75% (95% CI = 72.54-77.73) in men and 91% (95% CI = 88.66-92.13) in women (**Table 2**). The detailed site-specific prevalence of pain is shown in Table S1 in the **Online Supplementary Document**. We further classified the participants in five categories of age group (20 to 30, 31 to 40, 41 to 50, 51 to 60 and more than 60 years). Prevalence of PBE increased with increasing age for all the anatomical sites (**Table 3**, **Figure 2****,** and Table S2 in the **Online Supplementary Document**).

**Table 2:** Prevalence of pain at various anatomical sites* by sex (period January 2009 to January 2010, n = 2259)

Anatomical site	PBE in men (n = 1101)			PBE in women (n = 1158)			Difference in prevalence	
	**n**	**% Prevalence in male**	**95% CI**	**n**	**% Prevalence in female**	**95% CI**	**Male-Female**	**95% CI**
**A) Back Pain**	726	66	(63.68)	991	86	(83.88)	-20	(-23, -16)
**Neck**	373	34	(31.37)	612	53	(50.56)	-19	(-23,-15)
**Thoracic**	388	35	(32.38)	491	42	40.45)	-7	(-11,-3)
**Low back**	655	59	(56.62)	930	80	(78.83)	-21	(-25,-17)
**B) Any extremity pain**	695	63	(60.66)	907	78	(76.81)	-15	(-19,-11)
**1) Superior extremity†**	516	47	(44.50)	608	53	(50.55)	-6	(-10,-2)
**2) Inferior extremity‡**	579	53	(49.55)	838	72	(67.75)	-20	(-34,-16)
**C) Any pain (back/extremities)**	828	75	(72.77)	1048	91	(89.92)	-15	(-18,-12)

PBE – pain in the back and extremities, CI – confidence interval

*Categories are multiple and overlapping.

†Any one of the shoulder, arm, elbow, forearm, wrist, hand + fingers, trapezius/scapula.

‡Any one of the hip/buttocks, groin, thigh, knee, leg/calf, ankle, heel, foot + digits.

**Table 3:** Age group specific prevalence of pain at various anatomical sites* (January 2009 to January 2010)

Site of pain	Age 20-30 (n = 678)		Age 31-40 (n = 516)		Age 41-50 (n = 471)		Age 51-60 (n = 301)		Age >60 (n = 293)		Trend value	
	**n**	**% prevalence**	**n**	**% prevalence**	**n**	**% prevalence**	**n**	**% prevalence**	**n**	**% prevalence**	**Z score**	***P*-value**
**A) Back pain**	417	62	398	77	383	81	259	86	260	89	13.03	<0.001
**1) Neck**	207	31	229	44	231	49	166	55	152	52	8.71	<0.001
**2) Thoracic**	200	29	192	37	198	42	134	45	155	53	9.29	<0.001
**3) Low back**	367	54	370	72	350	74	252	84	246	84	12.72	<0.001
**B) Limb pains**	368	54	352	68	358	76	261	87	263	90	12.95	<0.001
**1) Superior extremity†**	236	35	243	47	265	56	188	62	192	66	10.33	<0.001
**2) Inferior extremity‡**	305	45	302	59	318	68	245	81	247	84	12.58	<0.001
**C) Any pain (back /extremities**	471	69	427	83	418	89	280	93	280	96	14.14	<0.001

*Categories are multiple and overlapping.

†Any one of shoulder, arm, elbow, forearm, wrist, hand + fingers, trapezius/scapula.

‡Any one of hip/buttocks, groin, thigh, knee, leg/calf, ankle, heel, foot + digits.

### Number of painful sites

**Figure 3:**
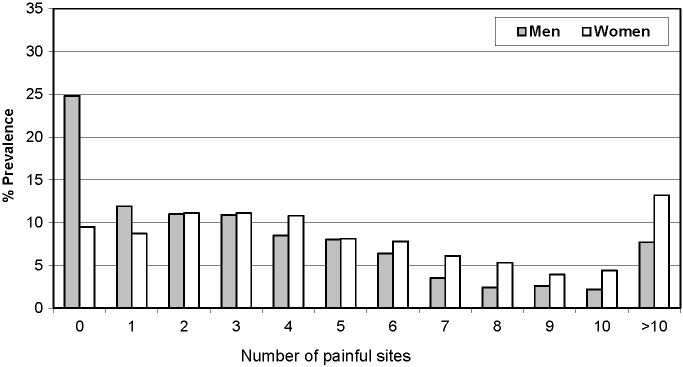
Number of painful sites per participant according to sex (period: 12 months, year 2009-10, n = 2259).

**Figure 4:**
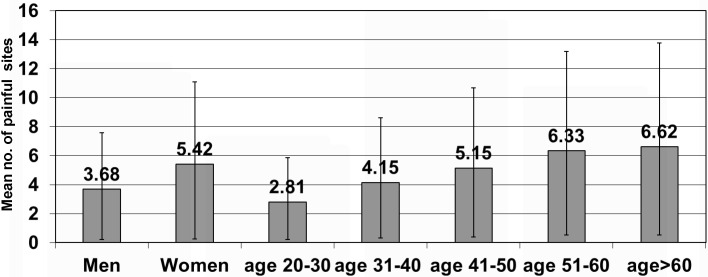
Mean number of painful sites per participant according to age and sex (January 2009 to January 2010, n = 2259).

The mean number of painful sites per woman participant 5.42 (95% CI = 5.17-5.67) was significantly higher than in men 3.68 (95% CI = 3.45-3.90). The mean number of painful sites per participant according to age also significantly increases from 2.81 (95% CI = 2.58-3.05) in the age group 20 to 30 years to 6.62 (95% CI = 6.10-7.14) in the age group of >60 years (**Figure 3****,** **Figure 4**, and Tables S3 and S4 in the **Online Supplementary Document**). The prevalence of 1 to 5 painful sites was comparable in men and women (50.3% and 49.8% respectively) but the prevalence of 6 to 10 painful sites was significantly higher in women (27.5%) than men (17.2%). The prevalence of zero painful sites was more than twice in men (24.8%) than women (9.5%), while the prevalence of more than 10 painful sites was almost twice in women (13.2%) compared to men (7.7%) (Table S5 in the **Online Supplementary Document**). Overall, both the prevalence as well as number of painful sites were higher in women and older age group suggestive of a sex and age-related gradient.

### Duration of pain

**Figure 5:**
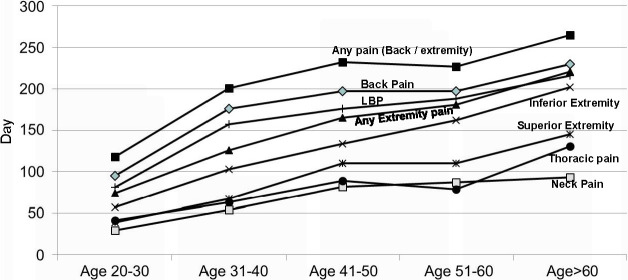
Mean duration of pain in different decades of life in symptomatic cases.

The mean duration of any pain per participant was significantly longer in women (203 days , 95% CI = 194.61-212.30) than men (127 days, 95% CI = 118.41-135.91) as well as in the participants of ≥50 years (232 days, 95% CI = 220.04-243.89), compared to the participants in the age group of 20 to 50 years (143 days, 95% CI = 135.56-150.11). This was consistent across all the main anatomical sites of the neck, thoracic, lower back, superior and inferior extremity (**Table 4****,** **Figure 5**).

**Table 4:** Sex and age specific duration of pain according to anatomical sites* (period January 2009-January 2010)

	Men				Women				20-50 years				>50 years			
	**Mean days per symptomatic (n = 828)**	**(95% CI)**	**Mean days per participant (n = 1101)**	**(95% CI)**	**Mean days per symptomatic (n = 1048)**	**(95% CI)**	**Mean days per participant (n = 1158)**	**(95% CI)**	**Mean days per symptomatic (n = 1316)**	**(95% CI)**	**Mean days per participant (n = 1665)**	**(95% CI)**	**Mean days per symptomatic (n = 560)**	**(95% CI)**	**Mean days per participant (n = 594)**	**(95% CI)**
**A) Back pain**	131	(122, 141)	99	(91, 107)	203	(194, 212)	184	(175, 193)	154	(146, 162)	121	(114, 128)	214	(201, 226)	201	(189, 214)
**Neck**	34	(28, 39)	25	(21, 30)	89	(81, 97)	81	(73, 88)	54	(48, 60)	43	(38, 47)	90	(78, 101)	85	(74, 96)
**Thoracic**	61	(54, 69)	46	(40, 52)	87	(79, 95)	79	(71, 87)	63	(57, 70)	50	(45, 55)	105	(93, 117)	99	(87, 111)
**Low back**	120	(110, 129)	90	(82, 98)	184	(175, 194)	167	(158, 176)	136	(128, 144)	107	(101, 114)	202	(189, 215)	190	(178, 203)
**B) Limb pains**	119	(110, 129)	90	(82, 98)	163	(154, 173)	148	(139, 157)	120	(112, 128)	95	(88, 101)	201	(188, 213)	189	(177, 202)
**1) Superior extremity†**	73	(65, 82)	55	(48, 62)	99	(90, 108)	90	(82, 98)	71	(64, 77)	56	(50, 61)	127	(115, 140)	120	(108, 132)
**2) Inferior Extremity‡**	95	(86, 104)	72	(64, 79)	143	(134, 152)	129	(121, 138)	96	(89, 104)	76	(70, 82)	182	(169, 195)	172	(159, 184)
**C) Any pain (back/extremities)**	169	(159, 179)	127	(118, 136)	225	(216, 234)	203	(195, 212)	181	(173, 189)	143	(136, 150)	246	(234, 258)	232	(220, 244)

CI – confidence interval

*Categories are multiple and overlapping.

†Any one of shoulder, arm, elbow, forearm, wrist, hand + fingers, trapezius/scapula.

‡Any one of hip/buttocks, groin, thigh, knee, leg/calf, ankle, heel, foot + digits.

Overall, the prevalence of acute (1 to 42 days) and chronic (more than 84 days) pain was significantly higher than the prevalence of sub-acute (43 to 84 days) pain in both men and women at all the anatomical sites. The prevalence of acute pain was higher in men than in women at all the anatomical sites (**Table 5**). In men, the prevalence of acute pain at neck and superior extremity (23% and 24% at respective sites) was higher than that of chronic pain (8% and 18% at respective sites) while the prevalence of acute (15%) and chronic (16%) thoracic pain was almost equal. In women, the prevalence of chronic pain was higher at all the anatomical sites. In the older participants, the prevalence of chronic pain was higher than acute pain at all the anatomical sites, whereas in the younger participants, specifically at neck (22%) and the superior extremity (22%), the prevalence of acute pain was more than chronic pain (14% and 18% at respective sites).

**Table 5:** Sex specific prevalence of pain according to duration at different anatomical sites*

	% prevalence of pain in study population of 1-42 days duration		% prevalence of pain in study population of 43 - 84 days duration		% prevalence of pain in study population of >84 days duration		% prevalence of pain in study population of 1-42 days duration		% prevalence of pain in study population of 43 - 84 days duration		% prevalence of pain in study population of >84 days duration	
	**Men (n = 1101)**	**Women (n = 1158)**	**Men (n = 1101)**	**Women (n = 1158)**	**Men (n = 1101)**	**Women (n = 1158)**	**Age 20-50 years (n = 1665)**	**Age >50 years (n = 594)**	**Age 20-50 years (n = 1665)**	**Age >50 years (n = 594)**	**Age 20-50 years (n = 1665)**	**Age >50 years (n = 594)**
**A) Back pain**	26	17	6	8	34	61	24	14	7	8	41	65
**Neck**	23	21	3	5	8	27	22	22	4	4	14	28
**Thoracic**	15	12	4	5	16	26	14	11	4	6	17	32
**Low back**	22	17	6	8	31	55	22	13	7	8	37	62
**B) Limb pains**	26	20	7	8	30	51	25	17	7	8	32	63
**1) Superior extremity†**	24	18	5	5	18	29	22	19	4	7	18	39
**2) Inferior extremity‡**	22	19	7	8	24	45	22	16	7	8	26	58
**C) Any pain (back/extremities)**	25	16	7	8	43	67	23	11	8	8	48	75

*Categories are multiple and overlapping.

†Any one of shoulder, arm, elbow, forearm, wrist, hand + fingers, trapezius/scapula.

‡Any one of hip/buttocks, groin, thigh, knee, leg/calf, ankle, heel, foot + digits.

### Intensity of pain according to sex and age and seasonality of pain

**Figure 6:**
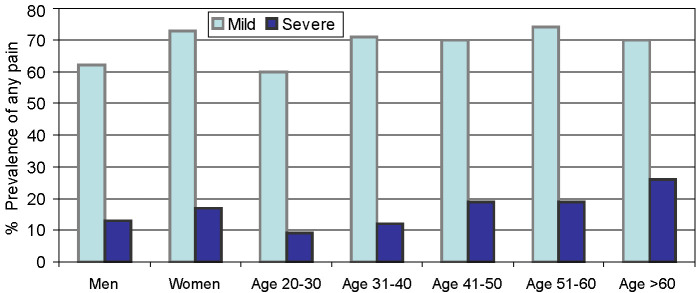
Age and sex specific intensity of any pain (January 2009 to January 2010, n = 2259). Categories are multiple and overlapping.

**Figure 7:**
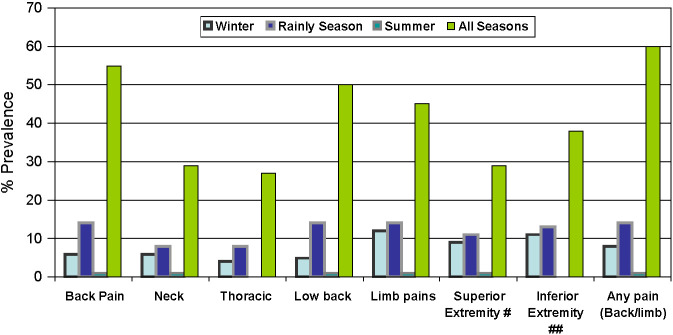
Season specific prevalence of pain at various anatomical sites. Categories are multiple and overlapping. #Any one of the shoulder, arm, elbow, forearm, wrist, hand + fingers, trapezius / scapula). ##Any one of the hip/buttocks, groin, thigh, knee, leg/calf, ankle, heel, foot + digits).

In both men and women, as well as participants across the age groups, the prevalence of mild pain was higher than severe pain at all the anatomical sites (**Figure 6**, Table S5 in the **Online Supplementary Document**). Similarly, for all the anatomical sites, the highest number of participants reported pain in all three seasons. Among those who reported pain during only a specific season, the prevalence of pain was higher in rainy season (**Figure 7**, Table S6 in the **Online Supplementary Document**).

## DISCUSSION

The five anatomical sites with the highest prevalence of pain, in both men and women as well as across the age groups were low back, knee, neck, leg and thoracic region. This may be due to higher load bearing activities of rural communities involving these sites. The high prevalence of neck pain can be due to the practice of carrying heavy load (firewood, earth) on head for long distance, especially by women.

The prevalence of acute pain was higher in men and younger participants than women and older for all the anatomical sites. The association between sudden unexpected maximum efforts such as bending, twisting, lifting with low back pain is known [11] and may be contributing to higher prevalence of acute back pain in men who are generally engaged in heavy manual labour. The higher prevalence of acute pain at neck (23%) and superior extremity (24%) in men, was probably due to heavy load bearing and digging. Therefore, specific interventions are needed to address the acute pain in this group. Apart from these exceptions, chronicity of pain was the norm corroborating with previously published literature [12]. Constant wear and tear of the bone and muscular frame because of physical hard labour, poor ergonomic postures, and certain nutritional factors may be the possible risk factors, but further studies are needed to identify precise causative pathways.

The prevalence of mild pain was higher than severe pain at all the anatomical sites. This is a hopeful sign, possibly suggestive of good coping mechanisms of the communities. Therefore, simple pain relief measures may yield results in reducing prevalence of PBE. The higher prevalence of low back pain, especially in the rainy season may be due to the bent position women adopt for long hours during planting saplings of paddy and harvesting as well as the heavy load carried by and manual work undertaken by men. Hence ergonomic interventions involving appropriate, low cost technology to reduce the physical burden of paddy cultivation or similar seasonal manual labour specific to the region may be explored as an intervention.

We compared the results from our study with those from other COPCORD studies. The higher prevalence of pain in low back than knee in both the sexes and in different age groups was similar to results of COPCORD studies from China [13], Indonesia [14], aborigines in Australia [15] and Bangladesh (8), but different from the findings of the Indian COPCORD series in which the prevalence of knee pain was higher than low back [7,9]. In general, women have a higher burden of acute and nonfatal chronic morbidities [16], especially temporary and persistent pain [17]. Our study also reported higher prevalence of pain in women at all the anatomical sites. This corroborated with other COPCORD studies [18-21], even though it differed from smaller set of studies reporting higher prevalence of back pain in men [22-24] or equal between sexes [25]. The highest prevalence of pain during the rainy season in our study differs from the Indian COPCORD study [19] in which the highest prevalence was in winter. This could be due to higher proportion of agrarian population in our study which is engaged in manual agricultural work the most during monsoon.

The factors affecting higher prevalence of PBE in our population would include higher prevalence of osteoporosis in women with increasing age compared to men [1]. This may have may have contributed to the higher prevalence of musculoskeletal pains in the women, especially in the older age group. Prevalence of pain at particular sites, especially at knee may be higher in women due to higher proportion of osteoarthritis, for which female sex, low body weight and lifting are known risk factors [26]. Nevertheless, this does not fully explain the difference in prevalence of knee pain between women and men, as the other risk factors such as heavy lifting and alcohol abuse [26] are also common in men. It has been reported that the causative factors of low back pain in African population differed from those in the Western population [27]. Similarly, therefore, the Indian population may have certain specific risk factors especially considering the less muscle mass and height as well as role of genetic factors [27].

To the best of our knowledge, this is the first study from rural India reporting in detail the population-based epidemiology of PBE including prevalence according to age and sex, the number of painful sites, duration, intensity as well as the seasonality of pain. The previous studies on epidemiology of PBE were primarily from peri-urban and relatively affluent rural areas [28], restricted to a certain type of labour population only, such as drill worker, cashew worker or jute worker [29-31], or were not population based [32]. However, it is possible that the epidemiology of PBE would vary according to different rural parts of India due to regional differences, the nature of occupational work communities are engaged in, socioeconomic status and access to care. In this aspect, the participants in this study can be considered more representative of the larger agrarian Indian communities with its significant dependence on manual labour.

This study, nested in the larger study to identify the overall 12 months period prevalence had several strengths which included random selection of the two study villages, high participation rate of the participants (89%) and data collection by CHWs with more than 15 years of experience using a well-tested, structured and robust questionnaire with rigorous quality control. These lend confidence to the estimates obtained. The key limitations of our study are the possibility of recall loss in reporting of pains from preceding 12 months and routine treatment of pains using aspirin by CHWs in these villages. Nevertheless, both these limitations would have resulted in underestimation and not overestimation of the burden of the problem.

## CONCLUSION

This population-based study from rural Gadchiroli describes the epidemiology of pain in back and extremities, identifies a significant gender and age related burden and underlines the chronic nature of the problem. It also provides clues to the different target populations (younger vs older and men vs women) for public health intervention. Further larger population based studies from different parts of rural India are needed to identify the regional estimates of the epidemiological variation in the profile of PBE as well as risk factors to understand the causation and explore intervention for PBE in rural Indian communities.

## Additional material


Online Supplementary Document
