# Supplementary material for: Epidemiology of pain in back and extremities in rural population: A community-based estimation of age- and sex-specific prevalence, distribution, duration and intensity of pain, number of painful sites and seasonality of pain during twelve months in rural Gadchiroli, India
Source: J Glob Health. 2021 Nov 27;11:12002. doi: 10.7189/jogh.11.12002 (PMC8647780; doi:10.7189/jogh.11.12002)
Supplement: Online Supplementary Document [file jogh-11-12002-s001.pdf]

Table S1. Prevalence of pain at various anatomical sites * by sex (Period January 2009 to January 2010, n=2259)

	Total (n=2259)		PBEin Male (n=1101)		PBEin Female (n=1158)		Difference in prevalence	
	n	% Prevalence	n	% prevalence in Male	n	% prevalence in Female	Male - Female	95% CI
**A) Back Pain**	1717	76	726	66	991	86	-19.64	(-23.09, -16.18)
**Neck**	985	44	373	34	612	53	-18.97	(-22.98,-14.95)
**Thoracic**	879	39	388	35	491	42	-7.16	(-11.16,-3.15)
**Low back**	1585	70	655	59	930	80	-20.82	(-24.15,-17.12)
**B) Limb pains**	1602	71	695	63	907	78	-15.20	(-18.9,-11.49)
**1) Superior Extremity #**	1124	50	516	47	608	53	-5.64	(-9.74,-1.51)
**Trapezius / Scapula**	255	11	105	10	150	13	-3.42	(-6 , -0.811
**Shoulder**	677	30	315	29	362	31	-2.65	(-6.42,1.12
**Arm**	547	24	236	21	311	27	-5.42	(-8.94,-1.89)
**Elbow**	324	14	117	11	207	18	-7.25	(-10.11,-4.38)
**Forearm**	261	12	80	7	181	16	-8.36	(-10.95,-5.766)
**Wrist**	468	21	179	16	289	25	-8.70	(-12.01,-5.388)
**Hand + Fingers**	277	12	114	10	163	14	-3.72	(-6.42,-1.03)
**Entire Superior Extermity**	46	2	10	1	36	3	-2.20	(-3.34,-1.05)
**2) Inferior Extremity ##**	1417	63	579	53	838	72	-19.78	(-23.69,-15.86)

**Buttocks / Hip**	286	13	75	7	211	18	-11.41	(-14.08,-8.73)
**Groin**	82	4	19	2	63	5	-3.71	(-5.22,-2.19)
**Thigh**	775	34	302	27	473	41	-13.42	(-17.28,-9.55)
**Knee**	1038	46	429	39	609	53	-13.63	(-17.7,-9.55)
**Leg / Calf**	881	39	326	30	555	48	-18.32	(-22.26,-14.37)
**Ankle**	478	21	153	14	325	28	-14.17	(-17.46,-10.87)
**Heel**	295	13	112	10	183	16	-5.63	(-8.38,-2.87)
**Foot + Digits**	240	11	75	7	165	14	-7.44	(-9.94,-4.93)
**Entire inferior Extermity**	40	2	8	1	32	3	-2.04	(-3.09,-0.96)
**C) Any pain (Back/limb)**	1876	83	828	75	1048	91	-15.30	(-18.35,-12.24)

* categories are multiple and are overlapping
# (Any one of Shoulder, Arm, Elbow, Forearm, Wrist, Hand + Fingers ,Trapezius / Scapula)
(Any one of Hip/Buttocks, Groin, Thigh, Knee, Leg / calf, Ankle, Heel, Foot + digits)

Table S2. Prevalence of pain at various anatomical sites* by age group (Period January 2009 to January 2010, n=2259)

Site of pain *	Total (n=2259)		Age 20 - 30 (n= 678)		Age 31 - 40 (n=516)		age 41 - 50 (n=471)		age 51 - 60 (n=301)		age >60 (n=293)	
	n	% Prevalence	n	% Prevalence	n	% prevalence	n	% prevalence	n	% prevalence	n	% prevalence
**A) Back Pain**	1717	76	417	62	398	77	383	81	259	86	260	89
**Neck**	985	44	207	31	229	44	231	49	166	55	152	52
**Thoracic**	879	39	200	29	192	37	198	42	134	45	155	53
**Low back**	1585	70	367	54	370	72	350	74	252	84	246	84
**B) Limb pains**	1602	71	368	54	352	68	358	76	261	87	263	90
**1) Superior Extremity #**	1124	50	236	35	243	47	265	56	188	62	192	66
**Trapezius / Scapula**	255	11	42	6	44	9	51	11	63	21	55	19
**Shoulder**	677	30	141	21	142	28	162	34	123	41	109	37
**Arm**	547	24	102	15	111	22	136	29	95	32	103	35
**Elbow**	324	14	35	5	70	14	97	21	65	22	57	19
**Forearm**	261	12	27	4	44	9	78	17	53	18	59	20
**Wrist**	468	21	83	12	85	16	123	26	94	31	83	28
**Hand + Fingers**	277	12	39	6	48	9	63	13	66	22	61	21
**Entire Superior Extermity**	46	2	3	0	8	2	10	2	11	4	14	5
**2) Inferior Extremity ##**	1417	63	305	45	302	59	318	68	245	81	247	84

**Buttocks / Hip**	286	13	32	5	60	12	69	15	61	20	64	22
**Groin**	82	4	8	1	14	3	19	4	18	6	23	8
**Thigh**	775	34	161	24	170	33	174	37	137	46	133	45
**Knee**	1038	46	159	23	200	39	250	53	207	69	222	76
**Leg / Calf**	881	39	199	29	187	36	193	41	151	50	151	52
**Ankle**	478	21	51	8	82	16	123	26	104	35	118	40
**Heel**	295	13	34	5	58	11	61	13	64	21	78	27
**Foot + Digits**	240	11	24	4	39	8	50	11	54	18	73	25
**Entire inferior Extermity**	40	2	1	0	6	1	8	2	10	3	15	5
**C) Back and limb pain both**	1443	64	314	46	323	63	323	69	240	80	243	83
**D) Only back pain**	274	12	103	15	75	15	60	13	19	6	17	6
**E) Only limb pain**	159	7	54	8	29	6	35	7	21	7	20	7
**F) Any pain (Back/limb)**	1876	83	471	69	427	83	418	89	280	93	280	96

* categories are multiple and are overlapping

(Any one of Shoulder, Arm, Elbow, Forearm, Wrist, Hand + Fingers ,Trapezius / Scapula)

(Any one of Hip/Buttocks, Groin, Thigh, Knee, Leg / calf, Ankle, Heel, Foot + digits)

Table S3. Mean and median number of painful sites per participant according to age and sex (Period January 2009 to January 2010, n=2259)

	Mean	SD	95 % CI	Median
**Total**	4.57	4.17	(4.40, 4.76)	4
**Sex**				
Men	3.68	3.70	(3.45, 3.90)	3
Women	5.42	4.35	(5.17, 5.67)	4
Difference in sex	-1.74		(- 2.07, - 1.40)	
**Age groups**				
age 20-30	2.81	3.08	(2.58,3.05)	2
age31-40	4.15	3.73	(3.83,4.47)	3
age 41-50	5.15	4.30	(4.76,5.54)	4
age 51-60	6.33	4.67	(5.80,6.86)	5
age>60	6.62	4.50	(6.10,7.14)	5
age group 20-50	3.89	3.79	(3.71, 4.07)	3
age > 50	6.48	4.60	(6.11, 6.85)	5

Table S4. Number of painful sites per participant (Period January 2009 - January 2010)

Number of painful sites	Total (n=2259)		Males (n=1101)		Females (n=1158)		Age group 20 - 50 (n=1665)		Age group >50 (n=594)	
	**n**	**%**	**n**	**% prevalence**	**n**	**% prevalence**	**n**	**% prevalence**	**n**	**% prevalence**
0	383	17.0	273	24.8	110	9.5	349	21.0	34	5.7
1	232	10.3	131	11.9	101	8.7	189	11.4	43	7.2
2	249	11.0	121	11.0	128	11.1	209	12.6	40	6.7
3	249	11.0	120	10.9	129	11.1	188	11.3	61	10.3
4	219	9.7	94	8.5	125	10.8	150	9.0	69	11.6
5	182	8.1	88	8.0	94	8.1	121	7.3	61	10.3
1 to 5	1131	50.1	554	50.3	577	49.8	857	51.5	274	46.1
6	161	7.1	71	6.4	90	7.8	122	7.3	39	6.6
7	110	4.9	39	3.5	71	6.1	76	4.6	34	5.7
8	87	3.9	26	2.4	61	5.3	57	3.4	30	5.1
9	74	3.3	29	2.6	45	3.9	45	2.7	29	4.9
10	75	3.3	24	2.2	51	4.4	41	2.5	34	5.7
6 to 10	507	22.4	189	17.2	318	27.5	341	20.5	166	27.9
> 10	238	10.5	85	7.7	153	13.2	118	7.1	120	20.2
Total	2259	100.0	1101	100.0	1158	100.0	1665	100.0	594	100.0

Table S5. Age and Sex specific intensity of pain at various anatomical sites*

(period January 2009 - January 2010, n=2259)

	Males (n=1101)		Females (n=1158)		20-30 (n=678)		31-40 (n=516)		41-50 (n=471)		51-60 (n=301)		above 60 (n=293)	
	**% Mild**	**% Severe**	**% Mild**	**% Severe**	**% Mild**	**% Severe**	**% Mild**	**% Severe**	**Mild %**	**Severe %**	**Mild %**	**Severe %**	**Mild %**	**Severe %**
**A) Back Pain**	50	16	67	19	51	10	63	14	61	20	63	23	59	30
**Neck**	29	5	47	6	26	4	39	5	43	6	49	7	43	9
**Thoracic**	28	8	34	8	25	5	33	4	30	12	35	10	41	12
**Low back**	45	15	62	18	45	9	58	14	54	20	61	23	56	28
**B) Limb pains**	56	7	68	11	49	5	62	6	66	10	74	12	72	18
**1) Superior Extremity #**	43	4	48	5	32	3	44	3	51	5	57	5	56	10
**2) Inferior Extremity ##**	46	7	63	9	41	4	53	5	58	9	70	11	67	17
**C) Any pain (Back/limb)**	62	13	73	17	60	9	71	12	70	19	74	19	70	26

* categories are multiple and are overlapping

(Any one of Shoulder, Arm, Elbow, Forearm, Wrist, Hand + Fingers ,Trapezius / Scapula)

(Any one of Hip/Buttocks, Groin, Thigh, Knee, Leg / calf, Ankle, Heel, Foot + digits)

Table S6. Season specific prevalence of pain at various anatomical sites*

(period January 2009 - January 2010, n=2259)

	Winter (Nov. Feb.)		Rainly Season (July - Oct.)		Summer (March - June)		All 3 season	
	**n**	**% prevalence**	**n**	**% prevalence**	**n**	**% prevalence**	**n**	**% prevalence**
**A) Back Pain**	132	6	323	14	20	1	1242	55
**Neck**	133	6	184	8	16	1	652	29
**Thoracic**	91	4	173	8	5	0	610	27
**Low back**	117	5	324	14	13	1	1131	50
**B) Limb pains**	271	12	309	14	12	1	1010	45
**1) Superior Extremity** #	208	9	239	11	12	1	665	29
**2) Inferior Extremity** ##	247	11	292	13	10	0	868	38
**C) Any pain (Back/limb)**	180	8	315	14	15	1	1366	60

* categories are multiple and are overlapping

(Any one of Shoulder, Arm, Elbow, Forearm, Wrist, Hand + Fingers ,Trapezius / Scapula)

(Any one of Hip/Buttocks, Groin, Thigh, Knee, Leg / calf, Ankle, Heel, Foot + digits)
